# Effects of Lifelong Ethanol Consumption on Brain Monoamine Transmitters in Alcohol-Preferring Alko Alcohol (AA) Rats

**DOI:** 10.3390/brainsci3020790

**Published:** 2013-05-15

**Authors:** Pia Jaatinen, Maija Sarviharju, Noora Raivio, C. J. Peter Eriksson, Antti Hervonen, Kalervo Kiianmaa

**Affiliations:** 1 School of Medicine, University of Tampere, Tampere 33014, Finland; E-Mail: pia.jaatinen@uta.fi; 2 Department of Internal Medicine, Tampere University Hospital, Tampere 33521, Finland; 3 Department of Alcohol, Drugs and Addiction, National Institute for Health and Welfare, Helsinki 00271, Finland; E-Mails: maija.sarviharju@thl.fi (M.S.); noora.raivio@helsinki.fi (N.R.); peter.cj.eriksson@helsinki.fi (P.E.); 4 Department of Public Health, University of Helsinki, Helsinki 00100, Finland; 5 School of Health Sciences, University of Tampere, Tampere 33014, Finland; E-Mail: antti.hervonen@uta.fi

**Keywords:** aging, ethanol, dopamine, noradrenaline, 5-hydroxytryptamine, animal model

## Abstract

The purpose of the present study was to examine the combined effects of aging and lifelong ethanol exposure on the levels of monoamine neurotransmitters in different regions of the brain. This work is part of a project addressing interactions of aging and lifelong ethanol consumption in alcohol-preferring AA (Alko Alcohol) line of rats, selected for high voluntary consumption of ethanol. Intake of ethanol on the level of 4.5–5 g/kg/day for about 20 months induced only limited changes in the neurotransmitter levels; the concentration of noradrenaline was significantly reduced in the frontal cortex. There was also a trend towards lower levels of dopamine and 5-hydroxytryptamine (5-HT) in the frontal cortex, and towards a lower noradrenaline level in the dorsal cortex. Aging was associated with a decreased concentration of dopamine in the dorsal cortex and with a declining trend in the striatum. The levels of 5-HT in the limbic forebrain were higher in the aged than in the young animals, and in the striatum, there was a trend towards higher levels in older animals. The data suggest that a continuous intake of moderate amounts of ethanol does not enhance the age-related alterations in brain monoamine neurotransmission, while the decline in the brain level of dopamine associated with aging may be a factor contributing to age-related neurological disorders.

## 1. Introduction

Both aging and chronic ethanol consumption are associated with several deficits in the brain. The impairments of cognitive and motor function have been linked with a number of deleterious morphological and functional changes involving different areas of the central nervous system (CNS). The contribution of aging and chronic ethanol consumption to the development of deficits may be separate or combined. The interactions of aging and chronic ethanol exposure have, however, been addressed in relatively few studies. 

Age-related deficits in motor and cognitive function have been attributed to impairment of monoaminergic neurotransmission [1,2]. Several studies have demonstrated selective changes in the concentration of noradrenaline, dopamine and 5-HT in different areas of rat CNS during aging [3,4,5,6,7]. Studies conducted with the mutant zitter rats have demonstrated age-related degenerative changes in the different terminal areas of the ascending dopaminergic and 5-hydroxytryptaminergic neuronal systems [6,8]. Moreover, age-dependent decreases in the neuronal density of some noradrenergic projection areas in the forebrain have been observed in immunohistochemical studies [9]. Age-related decline in dopamine D_1_ and D_2_ receptors in the nigrostriatal and mesocorticolimbic systems as well as in striatal dopamine transporter density have also been observed [10,11,12]. The dopamine release mechanism in the striatum, however, seems to remain unaffected in aged animals [13].

Previous studies on the interactions of aging and chronic ethanol exposure suggests that the age-related decline in monoaminergic functions is not drastically enhanced by ethanol. Administration of ethanol for six weeks was not found to accentuate the age-related loss of dopamine D_2_ receptors [11]. Human type 2 alcoholics showed similar decline in the density of dopamine transporter as the controls [12]. A remarkable sensitivity to ethanol-induced degeneration was, however, detected in the locus coeruleus, the major source of noradrenergic projections to almost every region of the brain; locus coeruleus neuron numbers decreased significantly in AA and ANA rats after lifelong ethanol exposure, while no age-related locus coeruleus neuron loss was seen in the control groups up to 2 years of age [14,15]. Furthermore, ethanol consumption was found to reduce GABA_A_ receptor subunit expression in a selective manner [16].

The purpose of the present study was to examine further the combined effects of aging and lifelong exposure to ethanol on the levels of monoamine neurotransmitters in different regions of the brain. Since age-related changes in the metabolism and distribution of ethanol in the body can be a contributing factor to ethanol-induced damage, the levels of ethanol and acetaldehyde in the blood were also measured. This work is part of a project addressing the interactions of aging and lifelong ethanol consumption in the alcohol-preferring AA and the alcohol-avoiding ANA line of rats, selected for high and low ethanol consumption, respectively [14,15,16,17,18,19,20,21,22,23,24]. 

## 2. Material and Methods

### 2.1. Animals and Treatments

The animal model used and the protocol for ethanol administration have been described in detail in previous studies [22,23,24]. In this part of the study 15 male and 16 female rats of the alcohol-preferring AA line from generation F_67_, and 5 male and 5 female rats from the generation F_71_ were used. The rats were housed in group cages (4–5 animals per cage) under standard conditions with a free access to standard rat food (RM1(E)SQC, SDS, Witham, UK). The experimental procedures were approved by the Institutional Animal Care and Use Committee at Alko Group Ltd. (Helsinki, Finland). 

The AA rats from generation F67 were randomly assigned to the old ethanol group (*n* = 19) and the old control group (*n* = 12). Voluntary ethanol consumption of the animals was measured at the age of 3 months, and again at the age of 24 months, by offering a free choice between water and 10% (v/v) ethanol in individual cages for 3 weeks [22]. Between these free-choice periods, the ethanol group was given 10%–12% ethanol as the only source of fluid (10% ethanol for one month, 12% ethanol thereafter), while the controls were given water. Consumption of ethanol when it was available as the only source of fluid (forced ethanol consumption) was determined in both groups at the age of 23 months. The animals of the young control group came from generation F_71_ (*n* = 10). They had free access to food and water throughout the experiment. 

### 2.2. Determination of Concentrations of Ethanol and Acetaldehyde in the Blood

Concentrations of ethanol and acetaldehyde in the blood were measured before and after the administration of ethanol 1 g/kg i.p. (12% w/v in saline) in both the ethanol group and the control group at the age of 12 months. Blood samples of 50 µL were taken from the tip of the tail immediately before (0 min) as well as 60 and 120 min after administration of ethanol. The blood hemolysates were stored at −20 °C until analysis with headspace gas chromatography [25,26]. The rate of ethanol metabolism was also calculated [25].

### 2.3. Assay of Monoamines in Brain Tissue

At the age of 24 months, after a one-week ethanol-free washout period, the rats were decapitated under deep sodium pentobarbital anaesthesia. The brains were immediately removed from the skull and dissected on ice into the dorsal part of the cerebral cortex, the frontal cortex, the striatum, the limbic forebrain (containing tuberculum olfactorium, nucleus accumbens and septum), the hippocampus, the hypothalamus, and the cerebellum. The tissue samples were frozen on dry ice and stored at −75 °C. The concentration of noradrenaline, dopamine, and 5-HT were measured by high performance liquid chromatography (HPLC), as described in detail previously [27].

### 2.4. Statistics

The normally distributed data on body weights and ethanol consumption are expressed as mean ± SEM. The monoamine, ethanol and acetaldehyde concentrations are given as median (min, max). The overall differences in brain monoamine concentrations were analyzed by using the non-parametric Kruskal-Wallis test, followed by the Conover-Inman test (young control *vs.* old control, old control *vs.* ethanol). Student’s *t*-test was used to compare body weights and ethanol consumption between the old control and the ethanol-exposed group, while the concentrations of acetaldehyde and ethanol and the metabolism of these were tested with the Mann-Whitney *U* test.

## 3. Results and Discussion

### 3.1. Body Weight and Ethanol Consumption

At the beginning of the experiment the body weights of the groups were similar; 286 ± 19 g in the ethanol group and 256 ± 28 g in the control group. The body weights increased significantly over age. The rats in the ethanol group tended to be heavier than the controls at the age of 12 (447 ± 35 g and 349 ± 45 g, n.s.) and 23 months (521 ± 40 and 433 ± 52 g, n.s.).

Voluntary consumption of ethanol at the age of 3 or 24 months did not differ significantly between the control group and the ethanol group (Table 1). There was no difference in forced ethanol consumption, either. Ethanol consumption in the present cohort of animals was essentially similar to that described in our previous reports [22,23,24]. 

**Table 1:** Consumption of ethanol (g/kg/day) during different phases of the experiment.

Group (*n*)	Voluntary consumption Age 3 months	Forced consumption Age 23 months	Voluntary consumption Age 24 months
Control (8)	5.3 ± 0.8	5.0 ± 0.6	5.5 ± 0.7
Ethanol (15)	5.2 ± 0.4	4.6 ± 0.4	4.7 ± 0.4

Mean daily consumption of ethanol as g of absolute ethanol/kg of body weight ± SEM. Apart from the 3-week test periods at the beginning and at the end of the experiment, the ethanol-exposed group had 10%–12% ethanol as the only source of fluid, while the control group was given only water to drink.

### 3.2. Concentrations of Ethanol and Acetaldehyde

The blood concentrations of ethanol and acetaldehyde were significantly higher in the ethanol-exposed group than in the controls at time point 0 min, *i.e.*, before the administration of ethanol, because the rats in the ethanol group had consumed ethanol during the night before, and all ethanol and acetaldehyde had not been metabolized (Table 2). The rates of ethanol and acetaldehyde metabolism, which virtually are the same, because of the high efficiency of acetaldehyde oxidation [28], were significantly higher in the ethanol group than in the control group. 

**Table 2:** Concentrations and elimination rates of ethanol and acetaldehyde at 12 months of age in the controls and in the ethanol-exposed group.

Group	Ethanol (mM)				Acetaldehyde (µM)		
**(*n*)**	**0 min**	**60 min**	**120 min**	**Δ**	**0 min**	**60 min**	**120 min**
Control (7–8)	0 (0; 0.1)	20 (9.7; 31)	8.6 (1.5; 22)	9.6	0 (0; 0.3)	1.3 (1; 2.1)	1.2 (0.6; 2.1)
Ethanol (14)	0.3 (0; 6.9) **	21.2 (11; 31.3)	8.7 (2; 15.7)	12.2 *	1.4 (0; 8.4) ***	2.4 (1; 11.3) *	2 (0.6; 10.6)

* *p* < 0.05; ** *p* < 0.01; *** *p* < 0.001, relative to the control group, Mann-Whitney *U* test; median, (min; max) are given. Δ elimination rates of ethanol and acetaldehyde were both estimated from the change in the ethanol concentrations between the 60 min and 120 min measurements.

### 3.3. Concentrations of Monoamines

The concentrations of monoamine transmitters in different areas of the brain are presented in Table 3. As gender had no effect on the monoamine concentrations, results from the male and female rats were pooled together. Both aging and ethanol induced selective alterations in regional monoamine concentrations. Aging was associated with a reduced concentration of dopamine in the dorsal cortex and an increased concentration of 5-HT in the limbic forebrain. Significant ethanol-induced alterations were found only in the frontal cortex, where the concentration of noradrenaline was lower in the ethanol rats than in the control rats. 

### 3.4. Discussion

The present study addressed the interaction of aging and lifelong ethanol consumption on the CNS concentrations of monoamine neurotransmitters in the alcohol-preferring AA rats. The underlying idea was to test, whether chronic ethanol consumption enhances the age-related changes in the CNS, as has been hypothesized [29,30]. The AA rats consumed *ca.* 4.5–5 g of ethanol/kg/day for 20 months, and the rate of ethanol and subsequent acetaldehyde elimination were significantly increased in the ethanol consuming animals, probably as a result of chronic exposure to ethanol. The AA rats acquire a high level of voluntary ethanol intake in about three weeks, when given a free access to ethanol solution with water and food freely available [24]. In earlier studies, lifelong consumption of ethanol by AA rats has been found to increase the behavioral sensitivity to ethanol over age and to produce morphological changes in peripheral sympathetic neurons and cerebellar cortex, as well as molecular alterations in brain GABA_A_ receptor subunits [14,15,16,17,18,19,20,21,22]. 

**Table 3:** Concentrations of monoamines in different brain areas of young control, old control, and old ethanol-exposed AA rats.

		Young control		Old control		Old ethanol	
**Frontal Cortex**							
	Noradrenaline	3.88	1.97; 4.30 (9)	3.96	2.90; 4.52 (8)	3.26	2.57; 4.03 (13) *
	Dopamine	1.37	0.66; 2.19 (8)	1.56	0.45; 3.40 (8)	0.81	0.58; 2.22 (11)
	5-HT	1.06	0.53; 2.32 (9)	1.18	0.29; 2.01 (8)	0.78	0.42; 1.34 (13)
**Dorsal Cortex**							
	Noradrenaline	4.34	1.95; 5.80 (9)	3.28	2.83; 4.41 (8)	2.54	2.08; 5.07 (13)
	Dopamine	0.26	0.10; 0.64 (8)	0.10	0.04; 0.32 (8) ^#^	0.09	0.04; 0.36 (13)
	5-HT	0.24	0.15; 0.35 (9)	0.21	0.10; 0.39 (8)	0.19	0.12; 0.25 (13)
**Hippocampus**							
	Noradrenaline	4.33	3.18; 6.54 (9)	3.96	3.24; 5.07 (8)	3.67	3.00; 4.69 (13)
	Dopamine	0.13	0.07; 0.38 (8)	0.24	0.07; 0.33 (7)	0.22	0.07; 0.57 (12)
	5-HT	0.04	0.04; 0.05 (9)	0.05	0.03; 0.06 (7)	0.04	0.03; 0.06 (11)
**Striatum**							
	Noradrenaline	2.15	1.48; 2.82 (9)	1.95	1.37; 3.63 (8)	1.87	1.44; 2.41 (13)
	Dopamine	75.93	41.67; 90.00 (9)	64.30	50.51; 83.98 (8)	60.20	37.67; 75.54 (13)
	5-HT	0.17	0.08; 2.21 (7)	1.04	0.12; 2.21 (6)	1.32	0.18; 2.30 (10)
**Limbic Forebrain**							
	Noradrenaline	7.38	3.67; 8.28 (8)	7.70	5.93; 9.66 (8)	6.90	4.37; 8.54 (13)
	Dopamine	28.85	18.50; 41.49 (9)	27.44	23.91; 40.37 (8)	28.61	23.85; 36.70 (13)
	5-HT	3.10	1.38; 6.91 (9)	12.45	1.70; 13.12 (8) ^#^	8.93	2.14; 13.04 (13)
**Hypothalamus**							
	Noradrenaline	13.77	7.02; 20.62 (9)	13.47	8.74; 19.82 (8)	14.58	11.87; 19.19 (12)
	Dopamine	2.37	1.88; 4.23 (9)	2.74	2.11; 3.28 (8)	2.70	1.83; 3.98 (13)
	5-HT	3.23	1.74; 3.81 (8)	3.31	2.69; 6.53 (7)	3.42	2.36; 5.66 (12)
**Cerebellum**							
	Noradrenaline	2.39	1.36; 2.60 (9)	2.24	1.97; 3.01 (8)	2.55	1.88; 3.05 (13)
	Dopamine	0.04	0.02; 0.07 (9)	0.06	0.04; 0.06 (7)	0.05	0.04; 0.07 (12)
	5-HT	0.06	0.04; 0.09 (9)	0.06	0.03; 0.07 (8)	0.05	0.04; 0.07 (13)

The concentrations are given in nmol/g tissue, median (min, max). * *p* < 0.05, Old ethanol *vs.* Old control group; ^#^ *p* < 0.05, Old control *vs.* Young control group; 5-HT: 5-hydroxytryptamine.

Earlier studies on monoamine neurotransmitters have revealed higher brain levels of dopamine and noradrenaline in the AA rats than in the alcohol-avoiding ANA rats [24]. The dopaminergic systems in the AA and ANA rats do not, however, differ in their sensitivity to ethanol. In the present study, the lifelong ethanol treatment induced only limited changes in the monoamine neurotransmitter levels; the concentration of noradrenaline was significantly reduced in the frontal cortex. There was also a trend (n.s.) towards lower levels of noradrenaline in the dorsal cortex, as well as those of dopamine and 5-HT in the frontal cortex of the ethanol-exposed animals. These results are in line with our earlier findings based on a similar experimental protocol, showing that lifelong ethanol intake was associated with a 26%–30% decrease in the neuron number of the locus coeruleus, the major source of noradrenaline in the CNS [14,15]. In other studies it has been reported that treatment with ethanol for six weeks did not enhance the age-related loss of dopamine D_2_ receptors in rats, and that the age-related decline in the density of dopamine transporter in human type 2 alcoholics was similar to the controls [11,12]. These data may indicate that noradrenergic neurotransmission is more sensitive to ethanol-induced degeneration than the dopaminergic or 5-hydroxytryptaminergic ones.

The level of ethanol consumption reached in the present study was estimated to result in peak nocturnal blood ethanol levels of about 10–20 mM, which can be considered intoxicating [31,32]. The neurotoxicity of ethanol in this kind of continuous exposure to moderate concentrations of ethanol seems to be rather limited—both quantitatively and regionally. It is possible that an intermittent exposure to ethanol could have resulted in more extensive alterations in CNS monoamine neurotransmission, as repeated withdrawal phases have been suggested to be more deleterious to neurons than chronic ethanol exposure per se [33,34,35]. Intermittent ethanol exposure (5.8 g/kg/day four days a week for 5.5 months) was previously found to induce significant loss of noradrenergic neurons in rat superior cervical ganglion, while no neuron loss occurred in rats exposed to similar amounts of ethanol continuously [36]. Moreover, loss of locus coeruleus noradrenergic neurons was seen in aged rats (29–30 months old) after only 5 weeks of intermittent exposure to heavily intoxicating doses of ethanol [37]. On the other hand, formation of acetaldehyde adducts, possible mediators of ethanol-induced toxicity, was reported in the frontal cortex and cerebellum of AA and ANA rats given a continuous ethanol treatment similar to the present one [20]. Selective volume loss of the anterior superior cerebellar vermis was also found after lifelong, continuous ethanol exposure [19].

Aging was associated with a decreased concentration of dopamine in the dorsal cortex and with a declining trend in the striatum. The results are in line with earlier reports showing reduced concentrations of dopamine in the brain of aged rats, especially in the dopamine rich areas [3,4,5,7]. They are also consistent with investigations reporting age-related decline in dopamine D_1_ and D_2_ receptors [10,11]. These findings together support the view that a reduction in dopaminergic neuronal functions contributes to age-related neurological manifestations, e.g. movement disorders [1,38]. 

In the old control group, the concentration of 5-HT in the limbic forebrain was higher than in the young animals, and in the striatum there was a trend towards higher levels in the old controls. The data on 5-HT reported in the literature are, however, somewhat conflicting. In a study by Woods and Druse [4], an age-related increase in 5-HT turnover was observed, which is probably consistent with the present data. In contrast with these findings, reduced levels of 5-HT have been reported in several brain regions in aged rats [5,7]. Therefore, the role of 5-HT in the mechanisms of aging and the age-related impairment of CNS functions remain to be clarified.

There were no significant alterations with aging in the brain levels of noradrenaline. These data are consistent with the study by Míguez and others [5], where the concentration of noradrenaline remained unchanged with aging in several brain areas and was significantly reduced only in the pons-medulla. On the other hand, decreased levels of noradrenaline, as well as reduced density of noradrenergic axons and varicosities, have also been reported in aged rats [7,9]. 

## 4. Conclusions

The present study addressed the combined effects of aging and chronic ethanol consumption on brain monoamine neurotransmitters using the alcohol-preferring AA line of rats as a model. The results showed that lifelong, continuous consumption of mildly intoxicating amounts of ethanol induced only modest and regionally selective changes in the monoamine neurotransmitter levels. These results along with previous data suggest that chronic intake of moderate amounts of ethanol does not potentiate the age-related alterations in neuronal structure and functions, but ethanol-induced neuronal changes are essentially different from those associated with aging. Age-related decline in the brain dopamine concentration supported the idea of dopaminergic neuronal functions contributing to age-related neurological dysfunction, e.g., impaired control of movement and posture.

## Abbreviations

AA: Alko Alcohol, alcohol-preferring rat line
ANA: Alko Non-Alcohol, alcohol-avoiding rat line
CNS: central nervous system
HPLC: high performance liquid chromatography
i.p.: intraperitoneal
n.s.: non-significant
SEM: standard error of the mean
5-HT: 5-hydroxytryptamine
